# Improving access to insulin

**DOI:** 10.2471/BLT.21.020421

**Published:** 2021-04-01

## Abstract

On the centenary of insulin’s discovery, initiatives are being launched with a view to reshaping the global insulin production landscape. Andréia Azevedo Soares reports.

Elizabeth Pfiester remembers the first time she learned that she had type 1 diabetes. She was 4 years old and had been rushed to hospital with diabetic ketoacidosis (DKA), a life-threatening condition which can occur as a result of insulin deficiency.

“Symptoms of DKA include vomiting, stomach pain and confusion. I was experiencing all of those and was hospitalized for two weeks. It was a horrible experience and if I hadn’t gotten treatment and been started on insulin injections I would have died,” Pfiester says.

As traumatic as the experience was, Pfiester knows she was one of the lucky ones: a child with access to diabetes care. Living in the United States of America, her parents had health insurance that covered her needs. That is until she graduated.

“From the day of my graduation, I was no longer covered by my father’s insurance plan and without the support of my parents would have struggled to pay the US$ 800 required to cover the cost of my insulin and other supplies each month.”

Now the head of diabetes advocacy group, T1International, Pfiester is one of an estimated 40 million people worldwide living with type 1 diabetes, a chronic condition in which the pancreas produces little or no insulin by itself. Before the discovery of insulin, most people with type 1 diabetes died within months of disease onset.

The World Health Organization (WHO) estimates that an additional 60 million people have type 2 diabetes, which results from the body’s ineffective use of the insulin produced.

For people living with either form of the disease, without treatment there are significant health risks that include kidney damage, eye damage, heart disease and/or stroke. Nerve damage of the feet increases the risk of foot ulcers and infections and may necessitate amputations. These complications can be prevented by adequate control of blood glucose levels, which requires regular monitoring and insulin for all of the people with type 1 and many of the people with type 2 diabetes.

“Most people living with diabetes are in low- and middle-income countries and an estimated 1 in 2 people worldwide cannot get access to the insulin they need,” says Bashier Enoos, a technical officer working on medicines and health products for noncommunicable diseases at WHO.

One of the main obstacles to insulin access is the cost of the hormone, whether short-acting human insulin or more sophisticated and more expensive insulin “analogues”, that have been genetically modified to act faster (for example to help glucose metabolism at mealtimes) or slower (for example to get a person with diabetes through the night).

“An estimated 1 in 2 people worldwide cannot get access to the insulin they need.”

Bashier Enoos

Typically, insulin is packaged in 10 mL vials, each vial containing 1000 units, which at a rate of 40 units per day will last 25 days. Some people take more, some less, and those paying for insulin out of pocket will spend more or less as a result.

Those expenditures can run quite high. In Nigeria, for example, it costs around US$ 100 for a month’s supply of insulin, which, along with the cost of glucose test strips and syringes, accounts for roughly 30% of the average monthly middle-class income in that country.

On the centenary of insulin’s discovery, the public health community is stepping up efforts to reduce insulin prices, as part of a broader push to improve access to diabetes treatments and technologies.

How far insulin prices can come down is a matter of debate. A study published in the March 2018 issue of *BMJ Global Health* estimated that it may be possible to profitably manufacture insulin at prices of US$ 72 per year or less (around US$ 6 per month) for human insulin and US$ 133 per year or less for insulin analogues.

The leading producers of insulin – including Eli Lilly, Novo Nordisk and Sanofi, which between them account for around 90% of global annual insulin production – have long argued that the prices they charge reflect spending on research and development, manufacturing, packaging and marketing, but have been reluctant to share expenditure data.

“One of the challenges we have faced in trying to establish fair pricing for insulin is the lack of transparency regarding these expenditures,” explains Allison Colbert, a technical officer working on medicines pricing and affordability at WHO.

It was partly to elucidate such issues that WHO convened a meeting between industry stakeholders on 23 and 24 February. Held under Chatham House rules (free use of the information exchanged without attribution), the meeting was attended by 13 private sector companies.

“The meeting aimed at encouraging inputs, commitments, and contributions from the pharmaceutical and health technology product industries to support WHO’s efforts to improve access to medicines and health technology products for diabetes,” Enoos says, adding that it was also an opportunity to discuss the barriers to access for insulin and associated health products such as glucometers and test strips.

The meeting was part of preparations for an informal collaborative agreement called the Global Diabetes Compact, which is to be launched on the 100th anniversary of insulin’s discovery on 14 April 2021. “The Compact will bring together the public, private and philanthropic sectors with a view to encouraging the alignment and collaboration needed to support diabetes prevention, management and treatment,” Enoos says.

On the treatment side, the Compact will emphasize improving access to medicines and technologies especially in low- and middle-income countries. As part of that effort WHO is encouraging the establishment of global medicine price reporting for insulin and other essential diabetes medicines and technologies purchased by low- and middle-income countries.

 “By bringing prices out into the open, national procurement agencies can get a clearer idea of where the market is and what they should be paying,” Colbert says.

Parallel to these efforts, WHO is also encouraging the development of biosimilar insulin, which can be marketed by manufacturers once patents have lapsed, are invalid, or have never been enforced on originator brands.

The term biosimilar is used for copies of biological drugs which, because of their complex, organic nature and the manufacturing methods used to make them, are highly similar to the original reference product.

“Prioritizing insulin access is long overdue.”

Elizabeth Pfiester

According to Erika Satterwhite – chair of the biosimilar committee of the International Generic and Biosimilar Medicines Association, which represents biosimilar producers – production of human insulin has already begun in several countries, among them Brazil, China, India and the Russian Federation.

She notes, however, that few national biosimilar producers have sought regulatory approval outside their domestic markets, or approval based on internationally accepted standards, leaving many countries reliant on more expensive originator or branded medicines.

To address this issue, in November 2019, WHO launched a pilot project, inviting manufacturers of biosimilar human insulin to apply for prequalification, a service provided by WHO to ensure the quality, safety and efficacy of products allowing them to be listed as eligible for procurement.

Many low- and middle-income countries use WHO’s lists of prequalified products as a reference to select quality-assured health products for national procurement. So, by having their products prequalified, manufacturers would potentially gain access to markets worldwide.

However, as of early March 2021, none had yet submitted an expression of interest. According to Matthias Stahl, team lead of medicines assessments in the WHO prequalification unit, discussions are ongoing with potential applicants.

Satterwhite suggests that more might be done to move things along. “It might be helpful to engage more in outreach and awareness about the pilot project,” she says. She also suggests opening the invitation to include analogue forms of the drug.

Stahl concedes that the pilot invitation may be too narrow in scope and might elicit more submissions if broadened. “Pilot project documents are not static and can be expanded and revised if desirable,” he says.

Watching from the sidelines, Pfiester is cautiously optimistic about these developments.

"We are glad that WHO is working on this, but prioritizing insulin access is long overdue. This centenary should be about celebrating an extraordinary breakthrough in science, instead we will spend it advocating for improved access to insulin.”

Pfiester now lives in the United Kingdom of Great Britain and Northern Ireland, where she moved in 2011 and finally got unfettered access to the drug she needed.

"I’d been worrying about my insulin and health-care costs for over a year, despite having two jobs,” she says. “It wasn't until I arrived in London that it fully hit me. I went to the pharmacy for the first time to pick up my medicines and I didn't have to pay anything. It’s something I will never forget. And I want everyone, regardless of income, location or status to know what that’s like.”

**Figure:**
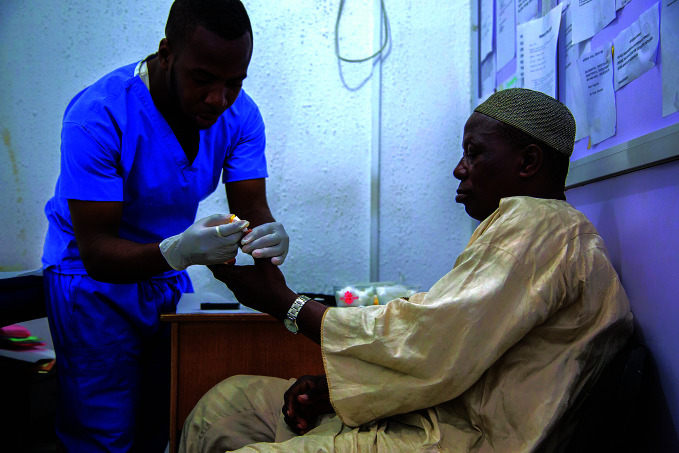
A health professional checks the blood glucose of a patient at the Rainbow Specialist Medical Center in Lekki, Lagos, Nigeria.

WHO/Andrew Esiebo

**Figure:**
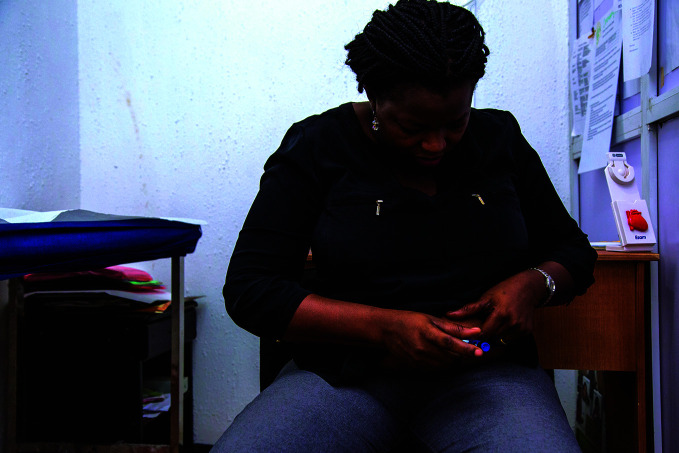
A person living with diabetes gives herself an insulin injection at the Rainbow Specialist Medical Center in Lekki, Lagos, Nigeria.

WHO/Andrew Esiebo

